# Manual Dexterity in Schizophrenia—A Neglected Clinical Marker?

**DOI:** 10.3389/fpsyt.2017.00120

**Published:** 2017-07-10

**Authors:** Maxime Térémetz, Loïc Carment, Lindsay Brénugat-Herne, Marta Croca, Jean-Pierre Bleton, Marie-Odile Krebs, Marc A. Maier, Isabelle Amado, Påvel G. Lindberg

**Affiliations:** ^1^FR3636, CNRS, Université Paris Descartes, Sorbonne Paris Cité, Paris, France; ^2^SHU, Resource Center for Cognitive Remediation and Psychosocial Rehabilitation, Université Paris Descartes, Hôpital Sainte-Anne, Paris, France; ^3^INSERM U894, GDR3557 Psychiatrie, Université Paris Descartes, Sorbonne Paris Cité, Paris, France; ^4^Service de Neurologie, Fondation OPH de Rothschild, Paris, France; ^5^Université Paris Diderot, Sorbonne Paris Cité, Paris, France

**Keywords:** schizophrenia, sensorimotor integration, manual dexterity, clinical marker, force control, independent finger movements

## Abstract

Impaired manual dexterity is commonly observed in schizophrenia. However, a quantitative description of key sensorimotor components contributing to impaired dexterity is lacking. Whether the key components of dexterity are differentially affected and how they relate to clinical characteristics also remains unclear. We quantified the degree of dexterity in 35 stabilized patients with schizophrenia and in 20 age-matched control subjects using four visuomotor tasks: (i) force tracking to quantify visuomotor precision, (ii) sequential finger tapping to measure motor sequence recall, (iii) single-finger tapping to assess temporal regularity, and (iv) multi-finger tapping to measure independence of finger movements. Diverse clinical and neuropsychological tests were also applied. A patient subgroup (*N* = 15) participated in a 14-week cognitive remediation protocol and was assessed before and after remediation. Compared to control subjects, patients with schizophrenia showed greater error in force tracking, poorer recall of tapping sequences, decreased tapping regularity, and reduced degree of finger individuation. A composite performance measure discriminated patients from controls with sensitivity = 0.79 and specificity = 0.9. Aside from force-tracking error, no other dexterity components correlated with antipsychotic medication. In patients, some dexterity components correlated with neurological soft signs, Positive and Negative Syndrome Scale (PANSS), or neuropsychological scores. This suggests differential cognitive contributions to these components. Cognitive remediation lead to significant improvement in PANSS, tracking error, and sequence recall (without change in medication). These findings show that multiple aspects of sensorimotor control contribute to impaired manual dexterity in schizophrenia. Only visuomotor precision was related to antipsychotic medication. Good diagnostic accuracy and responsiveness to treatment suggest that manual dexterity may represent a useful clinical marker in schizophrenia.

## Introduction

Although cognitive impairments predominate in schizophrenia (1, 2), sensorimotor abnormalities have also been noted since its very first description (3) and later on (1, 4–7). There is, however, no consensus on the relevance of these symptoms. More specifically, the following issues would need to be clarified: (i) can these symptoms be attributed to (antipsychotic) medication? (ii) do they reflect a primary genuine deficit of the underlying pathophysiology? and (iii) if they are genuine, could quantitative measurement of these dysfunctions serve as useful clinical markers of schizophrenia?

Extrapyramidal symptoms (8, 9) and other (upper limb) motor deficits (10, 11) have been attributed to antipsychotic pharmacotherapy, but this is being questioned and increasingly considered not to be the main cause of sensorimotor abnormalities in schizophrenia (7, 12, 13). Furthermore, sensorimotor control, rather than being impaired *per se*, has been viewed as being affected by abnormal cognitive function, such as deficient action planning (14–16).

However, evidence for genuine sensorimotor deficits in schizophrenia has been provided by investigating drug-naïve or differentially medicated subjects. Such deficits have been seen in psychomotor signs (17, 18), neurological soft signs (NSS) (19–22), postural control (23), micro-movements (24), eye movements (25–27), and upper limb control (28–31).

If sensorimotor impairments represent a primary deficit or co-vary with disease state, then measuring the degree of impairment could be clinically useful (32, 33), serve as a marker for vulnerability [e.g., Ref. (34, 35)] or describe neurodevelopmental abnormalities in schizophrenia (20, 36). However, validation of deficient dexterity as a clinical marker of schizophrenia (including assessment of sensitivity, specificity, and responsiveness) is lacking.

Here, we investigated manual dexterity in patients with schizophrenia, with the aim of probing its potential as a clinical marker. A high degree of manual dexterity (a hallmark of human upper limb use) requires efficient sensorimotor integration. Although different aspects of hand-use have been explored in schizophrenia, and often found to be deficient (30, 32, 37–39), quantifiable (rather than qualitative) measures of dexterity have rarely been studied.

We used the Finger Force Manipulandum (FFM) in visuomotor tasks (40) to quantify four manual dexterity components: precision of force control, motor sequence recall, timing during finger tapping, and independence of finger movements. We predicted that all of these components of dexterity would be deficient in schizophrenia patients compared to a healthy control group. Moreover, we hypothesized that some of these components would correlate with clinical outcome scores (such as NSS), but that each component would show correlations with specific clinical and neuropsychological scores. For example, we predicted that sensorimotor integration measured with NSS would correlate more closely to precision of force control than to motor sequence recall. Conversely, given that schizophrenia is associated with a core deficit in working memory (41), we also predicted that motor sequence recall would correlate best with disease status according to the Positive and Negative Syndrome Scale (PANSS). We investigated whether the degree of dexterity discriminated patients from controls, whether individual profiles of dexterous impairment could be extracted and, furthermore, how dexterity related to clinical and neuropsychological outcome, to antipsychotic medication as well as to responsiveness to treatment (cognitive remediation).

## Materials and Methods

### Participants

Thirty-five patients, 18–45 years of age, who met DSM-IV TR criteria (42) for schizophrenia were recruited at the Resource Center for Cognitive Remediation and Psychosocial Rehabilitation (C3RP), Sainte-Anne Hospital, Paris. Patients were clinically stabilized and under psychotropic medication for at least 1 month. Exclusion criteria: substance abuse/dependence, neurological disorders, participation in other cognitive remediation programs, resistance to neuroleptic treatment, electroconvulsive therapy in the previous 6 months, piano playing for a number of years.

Patients passed clinical and neuropsychological assessments, and comprehensive testing of manual dexterity. Twenty healthy age-matched subjects served as control group for the dexterity assessment. Table 1 lists clinical and demographic information. The study, approved by the local ethics committee (study no. 2011-A00454-37, Comité de Protection des Personnes, Ile de France 3), complied with the Declaration of Helsinki. Subjects provided written informed consent.

**Table 1:** Demographic and clinical characteristics of patients with schizophrenia and of control subjects.

	Patients (*N* = 35)	Control subjects (*N* = 20)

	Mean ± SD	Mean ± SD
**Demographic characteristics**		
Age (years)	31.2 ± 10.3	31.7 ± 9.6
Gender (male:female)	24:11	13:7
Education (years)	13.4 ± 2.5	16.9 ± 1.8
Moberg pick-up test (functional dexterity measure)	16.8 ± 7.6 s	12.0 ± 2.2 s
**Clinical characteristics**		
Age at first episode (years)	21.2 ± 6.4	
Disease duration (years)	12.1 ± 9.1	
Age of first antipsychotic treatment (years)	21.4 ± 5.3	
Age of first hospitalization (years)	23.6 ± 6.8	
Number of hospitalizations	3.0 ± 2.5	
Simpson-Angus Extrapyramidal scale	1.05 ± 2.06	
Antipsychotic treatment		
Chlorpromazine equivalent (CPZe, mg/day)	431± 340	
Other pharmacological treatments	% of Patients	
Antidepressant	34	
Anxiolytic	26	
Anticholinergic	11	
Hypnotic/sedative	9	
Thymoregulator	6	

### Remediation Protocol

Cognitive remediation therapy (43–45), to alleviate dysexecutive impairments was completed by 15 patients. It lasted 14 weeks (40 sessions of 60 min each: 2 sessions/week in the C3RP, 12 sessions at home) and comprised exercises on executive functions including attention, cognitive flexibility, planning, and memory.

### Clinical and Neuropsychological Assessment

Clinical evaluation (Table 1) comprised PANSS (46), NSS (22), and the Simpson-Angus Scale for rating abnormal movements (47).

Executive Function (Table 2) was assessed by selective attention [D2 test (48)], working memory [digit and spatial span of Wechsler Adult Intelligence Scale-III, WAIS-III (49)], word processing speed (digit symbol-copy of WAIS-III), cognitive flexibility [Wisconsin Card-Sorting Test (WCST) (50, 51)], action planning [zoo map test (52)], problem solving [D-KEFS Tower Test (53)], and inhibition [Stroop (54, 55)]. The above neuropsychological tests have been shown to be reliable and valid in schizophrenia (i.e., with test–retest correlation *R* > 0.7 or Cronbach’s alpha > 0.7): selective attention, D2 test (56); WAIS-III (57); WCST (58); action planning, zoo map test (59); problem solving, D-KEFS Tower Test (60); Stroop (58).

**Table 2:** Patient group (*N* = 35): clinical and neuropsychological scores.

Clinical score	Patients (*N* = 35)	Pre-remediation (*N* = 15^i^)	Post-remediation (*N* = 15^i^)
	Mean ± SD	Mean ± SD	Mean ± SD
**PANSS^a^**			

Total score	65.5 ± 14.6	68.3 ± 10.3	55.3 ± 10.1**
Positive symptoms	11.5 ± 3.5	11.4 ± 3.4	10.9 ± 3.5
Negative symptoms	18.25 ± 5.2	18.9 ± 4.7	15.7 ± 4.7**
Disorganization symptoms	8.6 ± 2.1	10.2 ± 2.3	7.7 ± 1.8**
General symptoms	35.8 ± 10.3	38.0 ± 8.8	28.8 ± 6.9**

**NSS^b^**			

Total score	13.37 ± 8.49	9.0 ± 4.7	6.4 ± 2.8*
Sensory integration sub-score	1.20 ± 1.54	1.4 ± 1.6	0.9 ± 1.4
Motor coordination sub-score	2.16 ± 2.03	2.8 ± 2.3	1.9 ± 2.1
Motor integration sub-score	0.31 ± 0.62	0.7 ± 1.0	0.4 ± 0.5

**Neuropsychological test**			

**WCST?^c^**			
Total number of categories	4.85 ± 2.02	5.0 ± 1.6	5.4 ± 1.3
**D2^d^**			
GZ	397.81 ± 106.21	396.1 ± 135.6	457.9 ± 108.9**
*F*%	3.7 ± 4.35	2.4 ± 1.9	2.7 ± 2.5
KL	163.22 ± 31.90	166.5 ± 33.4	191.9 ± 51.8**
GZ-F	390.77 ± 81.27	407.5 ± 86.5	450.3 ± 106.8**
**WAIS-III^e^**			
Digit span total	8.33 ± 2.71	8.4 ± 2.9	9.1 ± 2.1
Spatial span total	8.69 ± 2.71	8.2 ± 2.6	9.1 ± 2.9
Digit symbol—copy	104.22 ± 50.98	106.1 ± 36.2	95.9 ± 40.4
**BADS^f^**			
Zoo map test v1 planific. Time	157.74 ± 163.74	141.2 ± 102.1	172.9 ± 243.0
Zoo map test v1 total score	4.13 ± 3.50	4.6 ± 3.4	5.5 ± 3.6
**D-KEFS Tower test^g^**			
Ratio time/disk moves	4.41 ± 3.64	3.2 ± 0.8	3.2 ± 1.3
Ratio number of moves/number of minimal moves	1.73 ± 0.63	1.6 ± 0.4	2.3 ± 2.4
**Stroop^h^**			
Ratio interference/denominator	55.13 ± 27.69	61.5 ± 26.6	39.6 ± 10.8*

*Scores before and after cognitive remediation therapy for a subgroup of patients (*N* = 15)*.

*^a^PANSS: Positive and Negative Syndrome Scale (46)*.

*^b^NSS: neurological soft signs (22): three NSS sub-scores were retained: motor coordination, motor integration, sensory integration*.

*^c^WCST: Wisconsin Card-Sorting Test (51)*.

*^d^D2: The D2 Test of Attention (48)*.

*^e^WAIS-III: Wechsler adult intelligence scale, third ed (49)*.

*^f^BADS: behavioral Assessment of Dysexecutive Syndrome (52)*.

*^g^D-KEFS Tower Test: Delis–Kaplan Executive Function System (53)*.

*^h^Stroop: The Stroop Neuropsychological Screening (54, 55)*.

*^i^Not all 15 patients completed all listed tests pre- and post-remediation*.

*N = 14 for WCST, D2, WAIS-III*.

*N = 13 for BADS, D-KEFS*.

*N = 11 for NSS*.

*N = 10 for Stroop*.

*Significant differences between pre- and post-remediation at P < 0.5, **at P < 0.01 (Wilcoxon signed-rank test).

### Manual Dexterity Components

Finger movements were measured with the FFM,^1^ as previously described (40). Individual forces (of the index, middle, ring, and little finger) were sampled to a CED1401 (10 kHz sampling rate/digit) under Spike2v6,^2^ which provided real-time visual display of digit forces, target instructions or target forces. The Moberg pick-up test (61) gave a complementary functional measure of dexterity.

### FFM Tasks

**Figure 1:**
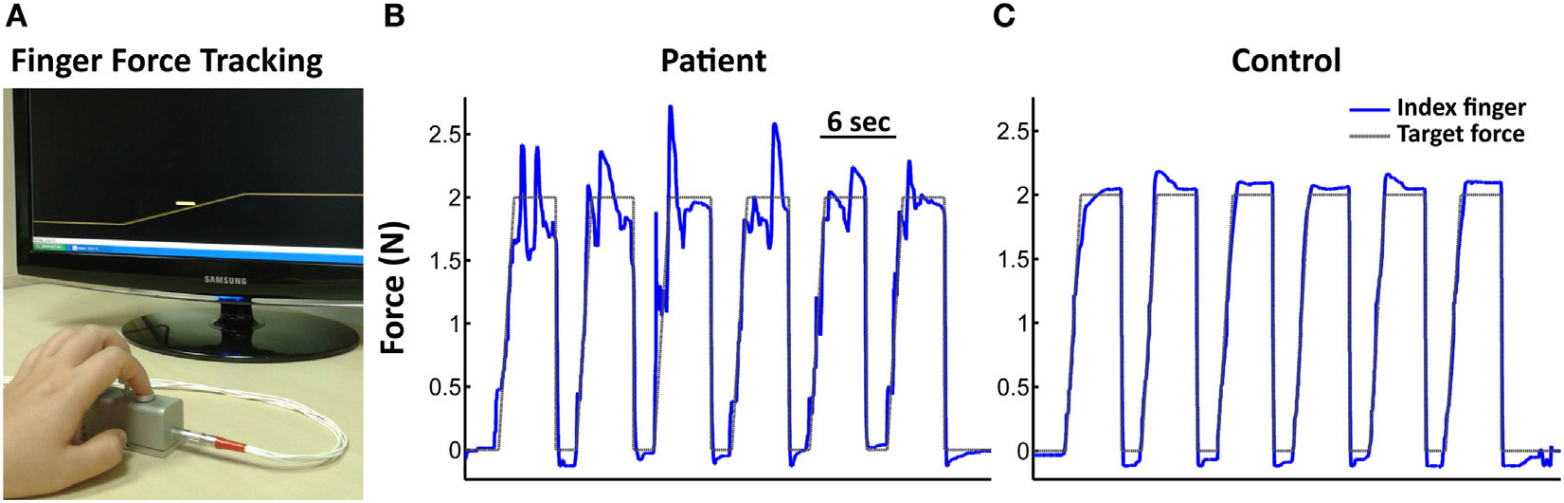
Finger Force Manipulandum (FFM) setup and single subject force-tracking examples. **(A)** FFM with a screen providing visuomotor feedback during the finger force-tracking task. The yellow line on the screen represents the target force. The cursor (horizontal bar close to the ramp) represents the instantaneous force exerted by the index finger. The subject had to match the vertical cursor position to the right-left scrolling target force. Target force represents a ramp-hold-and-release paradigm. Target forces of 1 N or 2 N hold-level correspond to a typical range employed in daily object manipulation. Subjects performed the task separately with the right index and the middle finger. **(B,C)** Single subject force-tracking examples of six successive trials at a target force (black trace) of 2 N with the index finger (blue trace). **(B)** For a patient. **(C)** For a control subject. Note greater tracking error in the patient.

(i) *Finger force tracking* (Figure 1A) was used to measure the ability to precisely control fingertip forces. By varying the force on the piston with the finger, the subject controlled a cursor on a computer screen and was instructed to follow the target force as closely as possible. Each of the 48 trials (eight blocks of six trials, four blocks with 1 N, four with 2 N target force) consisted of a ramp-hold-and-release trajectory, followed by a resting-phase.
(ii) *Sequential finger tapping* was used to assess the ability to learn and recall finger movement sequences. It consisted of a 5-tap finger sequence involving the four digits. The subject was instructed by sequential visual cues to press the indicated piston as soon as the target appeared. Each of three different sequences (A, B, C) was repeated 10 times with visual cues (learning trials), and then repeated five times (trials without cues) from memory and as quickly as possible (recall).
(iii) *Single-finger tapping* was used to test the performance of repetitive finger tapping at 1, 2, and 3 Hz. After an initial tapping period (15 taps, with auditory cues) the subject was instructed to continue tapping at the same rate for a similar period, without auditory cues.
(iv) *Multi-finger tapping* was used to quantify the independence of finger movements. Subjects were instructed to reproduce different finger tap configurations following a visual cue. The configurations varied trial-by-trial (pseudo-randomized) and consisted of one-finger taps (separate tap of index, middle, ring, or little finger) and two-finger configurations (simultaneous index-middle, index-ring, index-little, middle-ring, middle-little, or ring-little finger taps).

### Data Analysis

Task performance was analyzed using MatlabV7.5 (MathWorks Inc., Natick, MA, USA). Force signals were smoothed (5 ms sliding window) and down-sampled to 100 Hz. The following task-specific performance measures were extracted trial-by-trial.

(i) Finger force tracking: (48 trials)
  • Tracking error: root-mean-square error between applied and target force (separately extracted during ramp and hold).
  • Force onset: force onset time relative to the target ramp onset.
  • Release onset: onset time of force release relative to the end of the hold-phase.
  • Release duration: time taken to abruptly reduce the applied force from 75 to 25% of the target force.
  • Coefficient of variation (CV): SD/mean of force during 3 s of the hold-phase.
    For the three tapping tasks, a peak detection algorithm identified timing (occurrence), amplitude (>0.5 N) and origin (finger) of each tap. Subsequently, all taps were categorized as either correct (detected tap = required target tap) or incorrect (detected tap ≠ target tap). Incorrect trials consisted of “overflow taps” (presence of unwanted taps in non-target fingers while correctly tapping with the target finger) and “error taps” (presence of task-irrelevant taps in absence of a target finger tap). Subsequently, the following task-specific performance variables were calculated:
(ii) Sequential finger tapping: (45 trials). Two measures were computed for each 5-tap sequence:
  • Number of correct taps among the required 5-tap sequence (unwanted extra-finger-taps or incorrect taps were neglected).
  • Trial duration: period between the first and last tap of the required error-free sequence.
    Each measure was averaged over trials and conditions. A “sequence recall score” was computed expressing the average number of correct taps during recall.
(iii) Single-finger tapping: target finger taps were distinguished from taps in non-target fingers and the following measures calculated:
  • Tap interval: interval between two successive target finger taps.
  • Tap delay: time delay between the auditory signal and the target finger tap.
  • Number of overflow and error taps (see above)
(iv) Multi-finger tapping (64 trials):
  • Each trial was classified as correct or incorrect (presence of overflow taps and/or absence of target taps) and % correct taps was calculated (providing a measure of the degree of finger individuation).
  • Dual-tap interval: delay between the taps of the two fingers during trials requiring simultaneous two-finger tap configurations.

Respective measures were averaged/summed across trials and conditions.

### Statistical Analysis

Student’s *t*-test and Mann–Whitney *U*-test were used to test for group differences in parametric and non-parametric single-level variables. FFM measures were analyzed using repeated measures ANOVAs (Table 3). To extract individual profiles of dexterity we first selected the most discriminant “key-FFM” score (Table 3) in each task from group comparisons and then calculated individual *z*-scores for each key-FFM score, based on the control group’s performance: a *z*-score > 2 (>mean + 2SD, one-tailed) was considered out of normal range (i.e., a deficient score). A composite score (sum of the four key-FFM *z*-scores) was used for computing a receiver-operating curve to discriminate patients from control subjects based on the degree (or severity) to which dexterity was affected.

**Table 3:** Key measures of manual dexterity: relevant task-performance variables examined by ANOVA in each of the four FFM tasks.

ANOVA	Finger Force Manipulandum (FFM) task			
	Force tracking	Single-finger tracking	Sequential finger tapping	Multi-finger tapping
Independent variables	**Tracking error**	**Tap interval variability**	**Sequence recall score**	**Degree of finger individuation**
	Timing	Tapping rate	Number of correct taps	Dual-tap interval
	Release duration	Mean tap interval	Trial duration	
		Mean tap delay		
		Number of NLF taps		
Between-group factor	GROUP (patients, controls)	GROUP (patients, controls)	GROUP (patients, controls)	GROUP (patients, controls)
Within-group factors (task conditions)	FINGER (index, middle)	FREQUENCY (1, 2, 3 Hz)	SEQUENCE (sequence A, B, C)	
	FORCE (1 N, 2 N)	FINGER (index, middle, ring, little)	PHASE (1st half learning, 2nd half learning, recall)	
	PHASE (Ramp, Hold)	PHASE (with cue, without cue)		

*Independent variables examined by ANOVA are given for each FFM task. In bold: the key-FFM score (one for each task), which was the most discriminant variable for differentiating the performance between groups (patients vs. normal subjects). Within-group factors in CAPITALS, with their respective levels in parenthesis. *Post hoc* tests were performed using Fisher LSD Test and FDR correction for multiple comparisons was applied (62)*.

Spearman’s rank order or Pearson’s correlations were used to investigate relations between key-FFM performance measures and clinical or neuropsychological scores, and antipsychotic medication. Remediation effects on clinical/neuropsychological scores were tested with the Wilcoxon test. Statistical analysis was performed under Statistica©^3^ and level of significance set to *P* < 0.05. In correlation tests, significance level was corrected for multiple comparisons according to Benjamini and Hochberg (62).

## Results

### Clinical and Neuropsychological Data

Patient and control groups were similar in gender and age (Table 1). However, patients were significantly slower than control subjects in performing the pick-up test (Table 1, *T* = 2.74, *P* = 0.008), suggesting a qualitatively decreased level of dexterity. Full clinical details and neuropsychological scores are given in Table 2.

### Group Comparisons: Precision of Visuomotor Tracking

**Figure 2:**
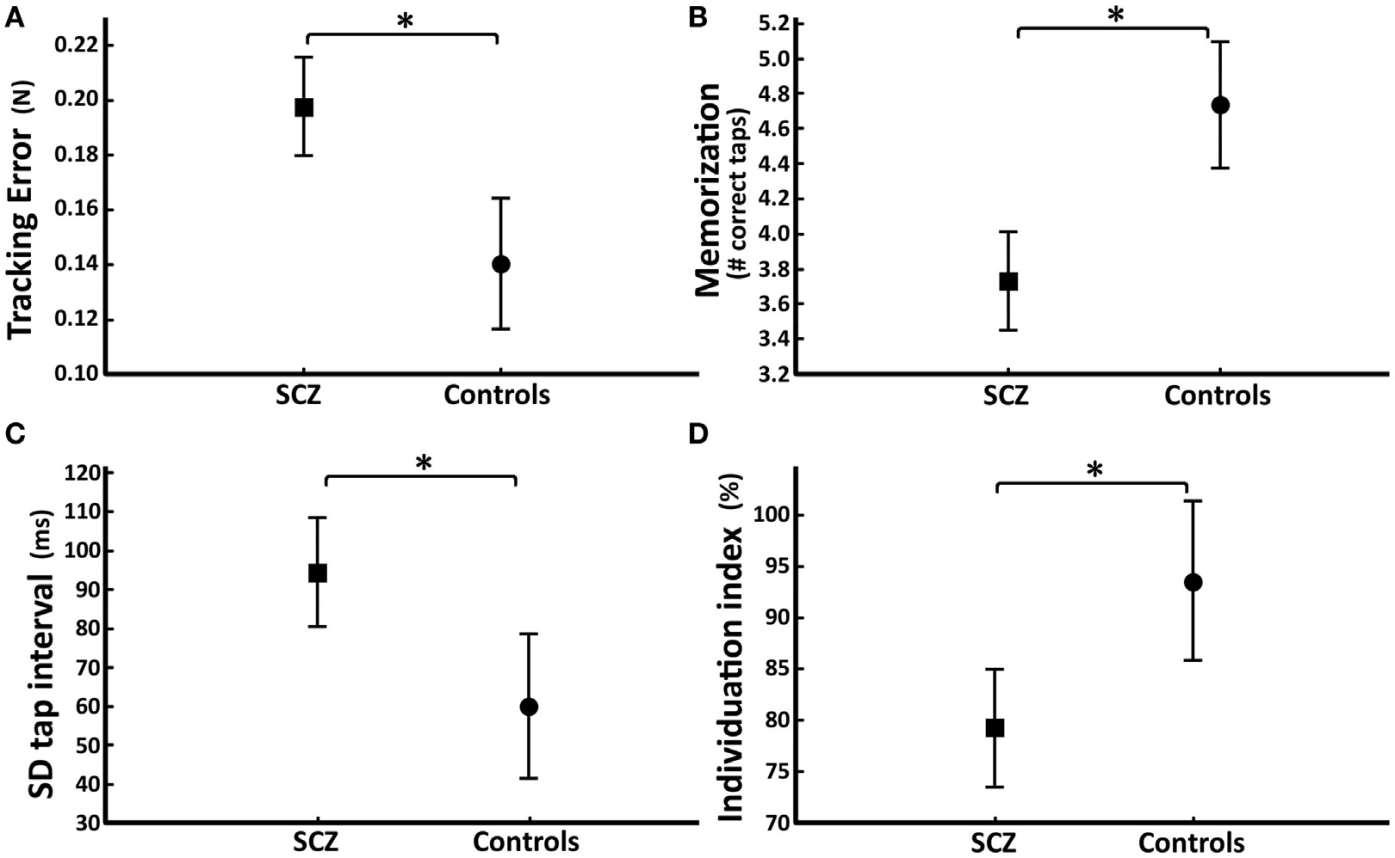
Group differences in the four key-Finger Force Manipulandum (FFM) scores. Average score (and SD) for each task. Patients (SCZ, represented by squares) vs. control subjects (circles). **(A)** Tracking error (N) during the finger force-tracking task. **(B)** Number of correct taps per trial during recall of the sequential finger-tapping task. **(C)** Tap interval variability (ms) across all conditions of the single-finger-tapping task. **(D)** Degree of individuation across all fingers for every combination during the multi-finger-tapping task. Compared to control subjects, patients with schizophrenia showed a statistically significant difference (*P < 0.05) in all four key-FFM scores.

Patients with schizophrenia showed altered finger force control during force tracking (Figures 1B,C). Patients had significantly higher tracking error compared to controls (SZ_patients: 0.20 ± 0.06 N; controls: 0.14 ± 0.04 N; GROUP effect: *F* = 14.9, *P* = 0.0003; Figure 2A) in all conditions (FORCES, PHASES). Force variability (CV) was also significantly increased in patients (SZ_patients: 2.86 ± 1.08; controls: 2.02 ± 0.56; GROUP effect: *F* = 4.6, *P* = 0.04). No significant differences were found in timing (force onset and release onset) or in release duration.

### Group Comparisons: Recall of Tapping Sequence

**Figure 3:**
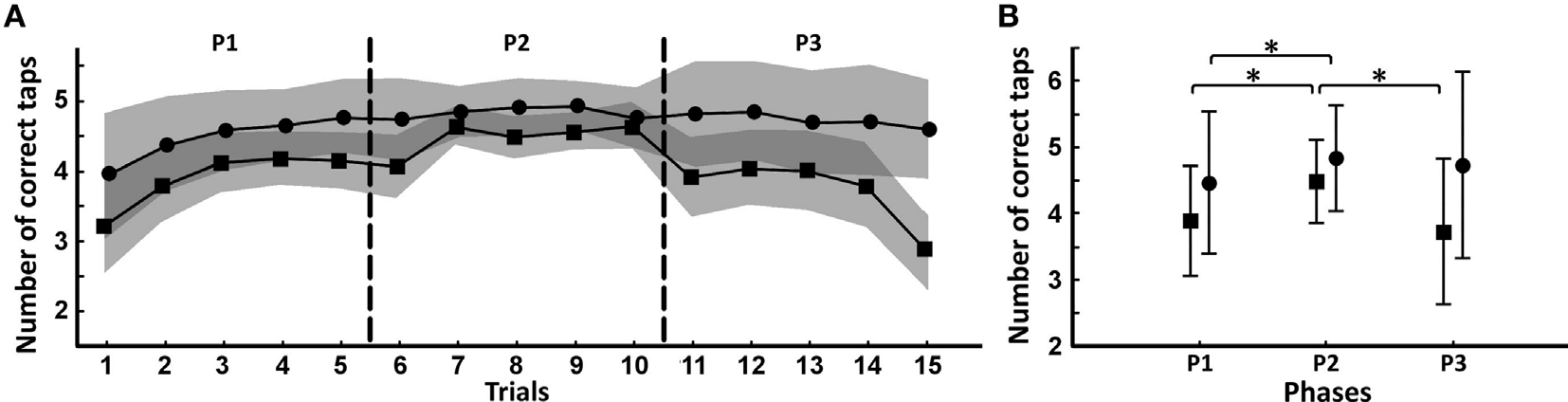
Evolution of performance during the sequential finger-tapping task. **(A)** Mean number of correct taps per trial across the 15 trials for the patient group (squares) and for the control group (circles). Vertical stippled lines indicate the three successive phases of the task. The SE of each group is represented by a gray area around the mean values (dark gray: overlap). **(B)** Mean number of correct taps for each phase consisting of five trials (P1: first half of the learning phase, P2: second half of the learning phase, P3: recall phase) in patient group (squares) and control group (circles). Horizontal lines represent within-group comparisons between P1/P2/P3. *Significant difference *P* < 0.05.

Patients (*N* = 33; two patients were excluded due to data acquisition problems) showed a significantly decreased number of correct taps over the entire task compared to controls (SZ_patients: 4.04 ± 0.63; controls: 4.68 ± 0.25; GROUP effect: *F* = 18.9, *P* = 0.00007). This difference was even greater during recall (SZ_patients: 3.73 ± 1.00; controls: 4.74 ± 0.30; GROUP*PHASE effect: *F* = 6.47, *P* = 0.002; *post hoc* test P3: *P* < 0.000001; Figure 2B). During task progression, patients and controls increased the number of correct taps (TRIALS effect: *F* = 12.7, *P* < 0.000001; Figure 3A). When splitting the task into three consecutive phases [first (P1) and second (P2) half of the learning phase, and the recall phase (P3)], patients started the task with a significantly reduced number of correct taps compared to controls (SZ_patients: 3.89 ± 0.68; controls: 4.47 ± 0.48; GROUP/PHASE effect: *F* = 6.47, *P* = 0.002; *post hoc* test P1: *P* = 0.002; Figure 3B). Both groups increased their number of correct taps during the second learning phase (SZ_patients: 4.48 ± 0.56; *post hoc* test P1/P2: *P* = 0.000001; controls: 4.84 ± 0.20; *post hoc* test P1/P2: *P* = 0.013), and patients reached a similar success rate as controls (somewhat lower, but statistically non-significant, *post hoc* test P2: *P* = 0.06). During recall, controls maintained the same number of correct taps (controls: 4.74 ± 0.30; *post hoc* test P2/P3: *P* = 0.49) while the performance of the patients decreased to levels seen in the initial (P1) learning phase (SZ_patients: 3.73 ± 1.00; *post hoc* test P2/P3: *P* = 0.000001). Both groups made the same kind of errors during the task and patients had a significantly longer trial duration during recall compared to controls (SZ_patients: 2,674 ± 693 ms; controls: 2,027 ± 480 ms; GROUP effect: *F* = 12.9, *P* = 0.00001).

### Group Comparisons: Timing in Single-Finger Tapping

In the single-finger-tapping task, the average tap frequency was similar in patients and in control subjects, close to the 1, 2, and 3 Hz target frequency, during the cued as well as during the non-cued phase. However, patients showed a significantly increased tap interval variability compared to controls (SZ_patients: 94.5 ± 50 ms; controls: 60.1 ± 17.2 ms; GROUP effect: *F* = 8.8, *P* = 0.004; Figure 2C), and this difference was similar in all conditions (FINGER, FREQUENCY, PHASE). There was no group difference in other task measures (erroneous taps).

### Group Comparisons: Independence of Finger Movements

In the multi-finger-tapping task, patients showed a significantly reduced degree of finger individuation compared to controls (SZ_patients: 79 ± 20%; controls: 94 ± 6%; GROUP effect: *F* = 9.0, *P* = 0.004; Figure 2D). This was the case in all fingers and for single- or multi-finger tap configurations. Patients also had significantly longer dual-tap intervals (SZ_patients: 380 ± 18 ms; controls: 304 ± 24 ms; GROUP effect: *F* = 6.6, *P* = 0.01).

### Heterogeneity of Dexterity Profiles

**Figure 4:**
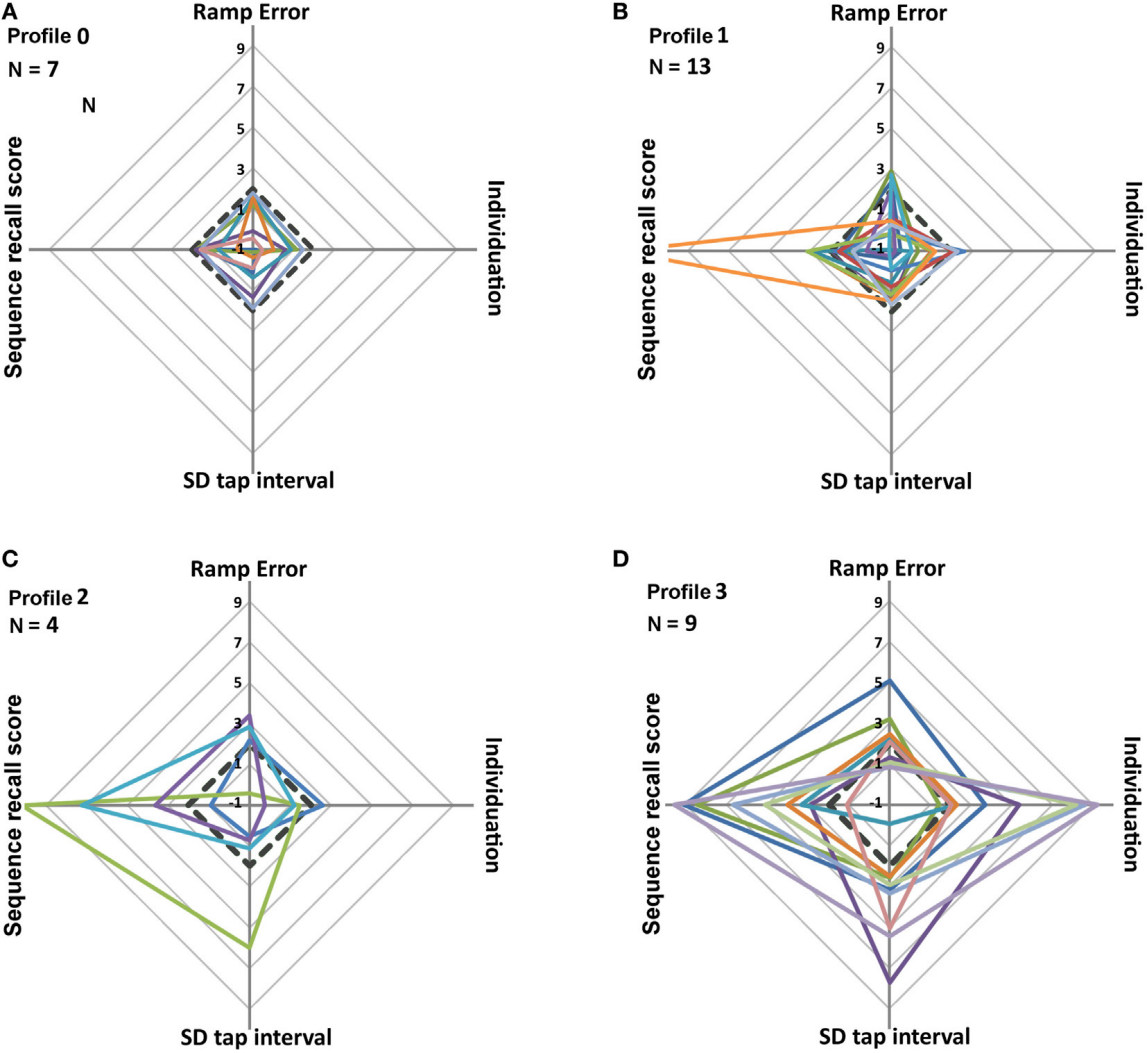
Radar plots of the four key-Finger Force Manipulandum scores (ramp error in finger force tracking, degree of individuation in multi-finger tapping, tap interval variability in single-finger tapping, and sequence recall index in sequential finger tapping). Each measure represents a *z*-score relative to the mean and SD of the control group. The 33 patients were subdivided into four types of dexterity profiles **(A–D)** according to the number of affected *z*-scores per patient. **(A)** Profile_0 (all *z*-scores <2). **(B)** Profile_1 (one *z*-score >2). **(C)** Profile_2 (two *z*-scores >2). **(D)** Profile_3 (three or four *z*-scores >2). Black dotted lines represent the normality threshold (mean + 2SD). Scores > threshold are considered abnormal.

Individual dexterity profiles are shown as radar plots of the four key-FFM measures (Figure 4). Four types of profiles were distinguished according to the number of affected *z*-scores in each patient. “Profile_0” (*N* = 7 patients) with all *z*-scores <2 (i.e., non-affected), “profile_1” (*N* = 13) with one affected *z*-score >2, “profile_2” (*N* = 4) with two affected scores, and “profile_3” (*N* = 9) with three or four affected *z*-scores. Sequence recall was the most frequently affected component (*N* = 16), followed by degree of individuation, ramp error, and tap interval variability. Profile_0 showed homogeneous radar plots (Figure 4A) with all *z*-scores <2 (below pathology threshold). In profile_1 the majority of the patients had affected *z*-scores close to threshold (3 > *z*-score > 2; Figure 4B). In profile_2 and profile_3 (Figures 4C,D), most of the affected *z*-scores were >3 or >4, resulting in heterogeneous, large amplitude patterns. Qualitatively, patients with a large number (>2) of affected scores also showed highly abnormal values (*z*-score > 3; Figure 4). Furthermore, dexterity profiles showed strong heterogeneity with only 15% of patients showing abnormal values in a common set of components.

### Low Covariance between Key-FFM Measures

The heterogeneity among dexterity profiles suggested that each key-FFM component represents a specific element of control, independent from the other components. A quantitative estimate of their independence was obtained by pair-wise Pearson correlations: this showed that only one among the six pair-wise correlations was statistically significant, i.e., tap interval variability vs. degree of individuation (*r* = 0.54, *P* < 0.05, Dof = 32). This lack of systematic covariance between key-FFM scores indicates a large (but not perfect) independence among component measures.

### Sensitivity and Specificity of Key-FFM Measures

**Figure 5:**
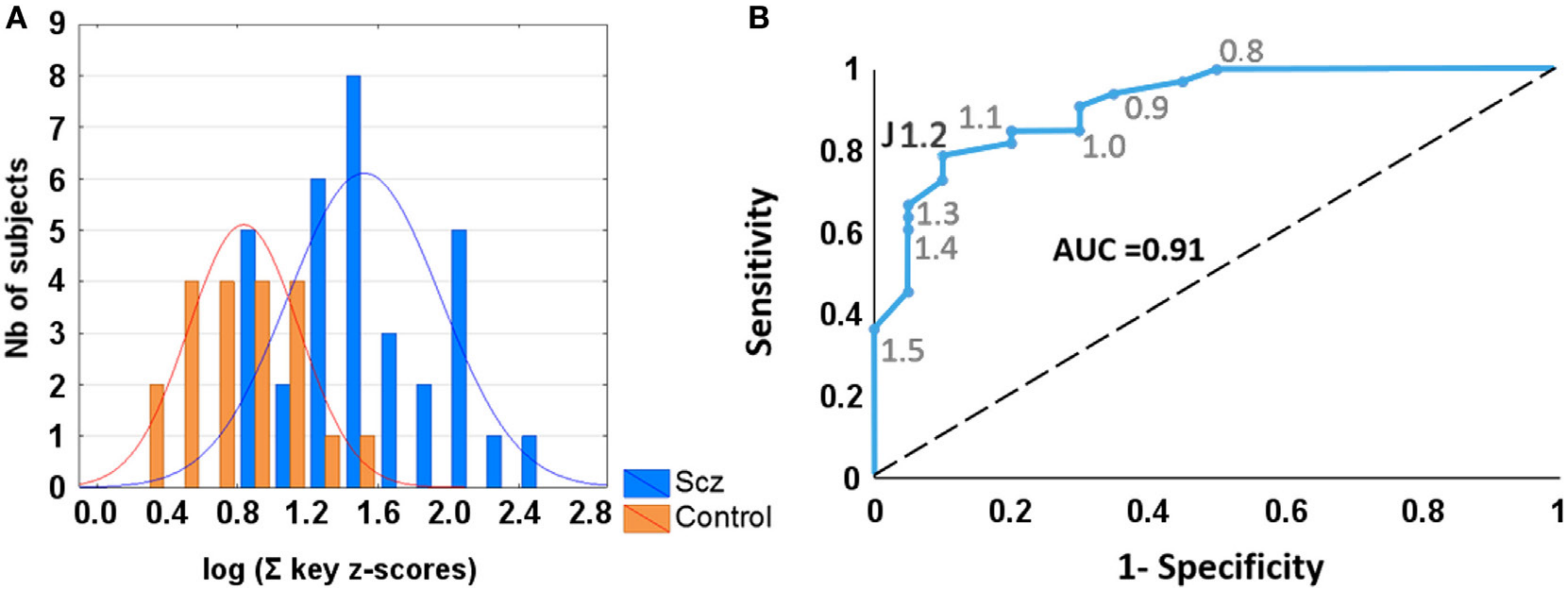
**(A)** Histograms of log-transformed summed key-Finger Force Manipulandum scores in patients with schizophrenia (blue vertical bars) and control subjects (orange bars). **(B)** Receiver-operating characteristic curve showing sensitivity and specificity of various criterion levels. Youden’s J statistic shows that the optimal value of 1.2 gives sensitivity = 0.79 and specificity = 0.9. Large area under curve (AUC = 0.91) suggests positive diagnostic interest of summed dexterity score.

The composite dexterity measure (sum of the four key-FFM scores) was analyzed using receiver-operating characteristics (ROC) to test for sensitivity and specificity (Figure 5). A log value of 1.2, corresponding to a score = 3.3, yielded best discrimination between patients and controls, with a positive predictive power = 0.93 and a negative predictive power = 0.9. In comparison, ROC analysis of each single key-FFM score provided lower discriminative power. Similarly, using the NSS total score as a means of discrimination (mean = 5 ± 2, threshold = 9, obtained from (22)) resulted in weaker positive predictive power (0.65).

### Relation between FFM Measures and Clinical Outcomes

**Figure 6:**
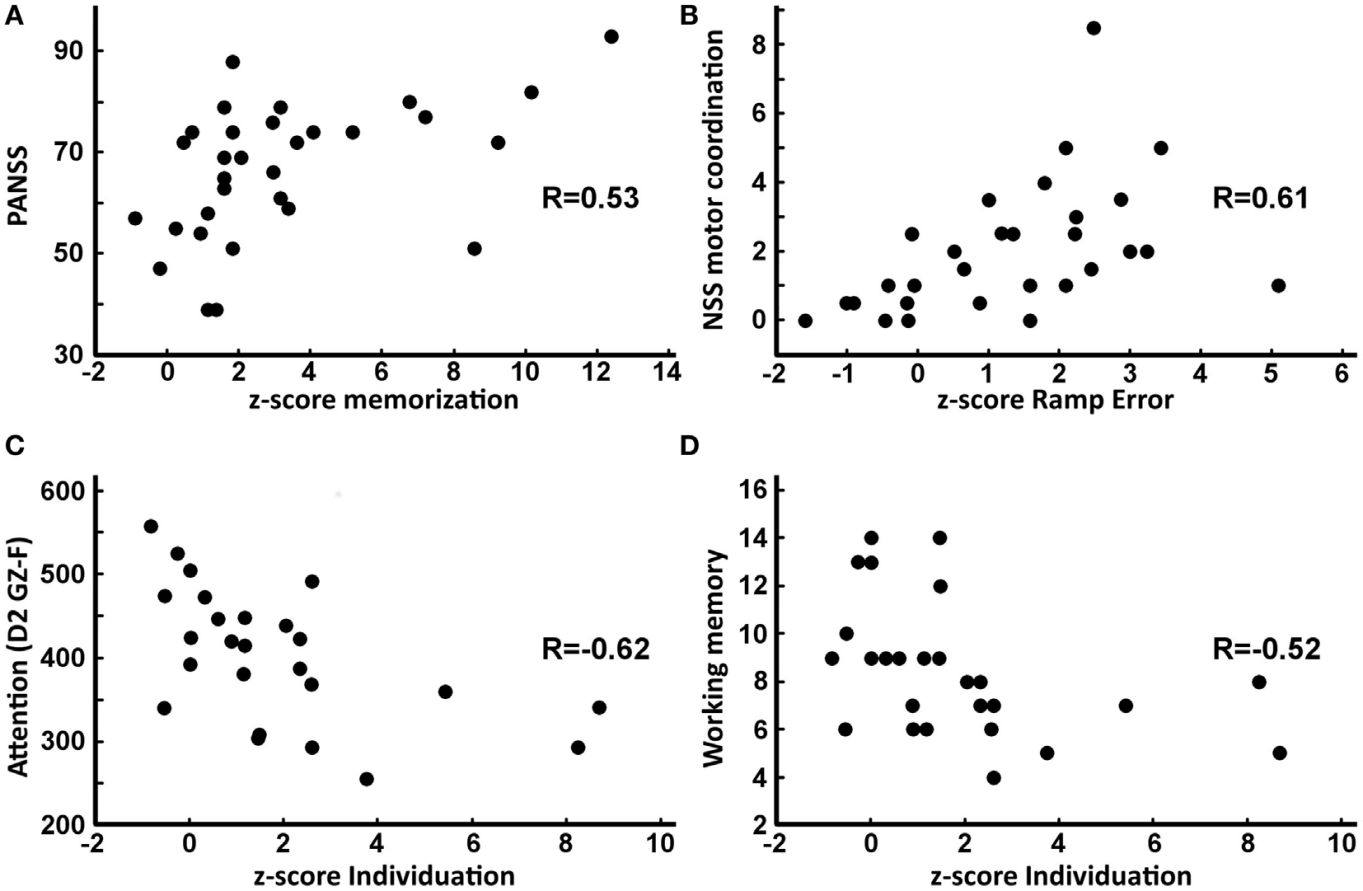
Relation (Spearman correlations) between key-Finger Force Manipulandum *z*-scores and clinical or neuropsychological scores. **(A)** Positive correlation between sequence recall score and total Positive and Negative Syndrome Scale (PANSS) score (*R* = 0.53, *P* = 0.0019). **(B)** Positive correlation between ramp error and motor coordination neurological soft sign (NSS) sub-score (*R* = 0.53, *P* = 0.003). **(C)** Negative correlation between degree of individuation and GZ-F D2 attention sub-score (*R* = −0.62, *P* = 0.0009). **(D)** Negative correlation between degree of individuation and working memory score (*R* = −0.52, *P* = 0.005).

For patients with schizophrenia, correlations were computed between key-FFM scores and clinical (PANSS; NSS) and neuropsychological (D2, WAIS-III) outcomes. Briefly, FFM sequence recall scores correlated with PANSS total scores (Figure 6A; *r* = 0.53) and with PANSS disorganization scores (*r* = 0.55, *P* < 0.05, Dof = 30). FFM tracking error correlated with NSS motor coordination (Figure 6B; *r* = 0.61), with NSS sensory integration (*r* = 0.52) and with NSS motor integration (*r* = 0.53, *P* < 0.05, Dof = 28). FFM tap interval variability did not show any correlations. FFM degree of individuation correlated with NSS sensory integration sub-score (*r* = 0.50, *P* < 0.05, Dof = 28), with different sub-scores of the D2 test of attention (Figure 6C; D2 GZ (*r* = −0.61), D2 KL (*r* = −0.63) and D2 GZ-F (*r* = −0.62), *P* < 0.05, Dof = 25), and with the WAIS-III working memory score (Figure 6D; *r* = −0.52, *P* < 0.05, Dof = 26). No relation was found between FFM measures and the scores of Stroop, D-KEFS Tower or BADS.

### Potential Influence of Medication

The chlorpromazine equivalent (CPZe) correlated positively with the composite dexterity measure (*r* = 0.50; *P* = 0.005; Dof = 28), but not with single key-FFM scores, except for ramp error (*r* = 0.47, *P* < 0.05, Dof = 28).

Additional multiple regression analyses showed that correlations between each of the three key-FFM scores (degree of individuation, tap interval variability, and sequence recall) with clinical and neuropsychological outcomes (Table 3) remained significant even with CPZe as a covariate, indicating that antipsychotic medication could not completely explain these relations. Furthermore, two different key-FFM scores (tap interval variability and individuation) were more affected in patients with prescribed anxiolytic medication (in 26% of patients), but not with mood stabilizers, antidepressants, or anticholinergic medication.

### Change of Dexterity and of Clinical/Neuropsychological Scores with Cognitive Remediation

**Figure 7:**
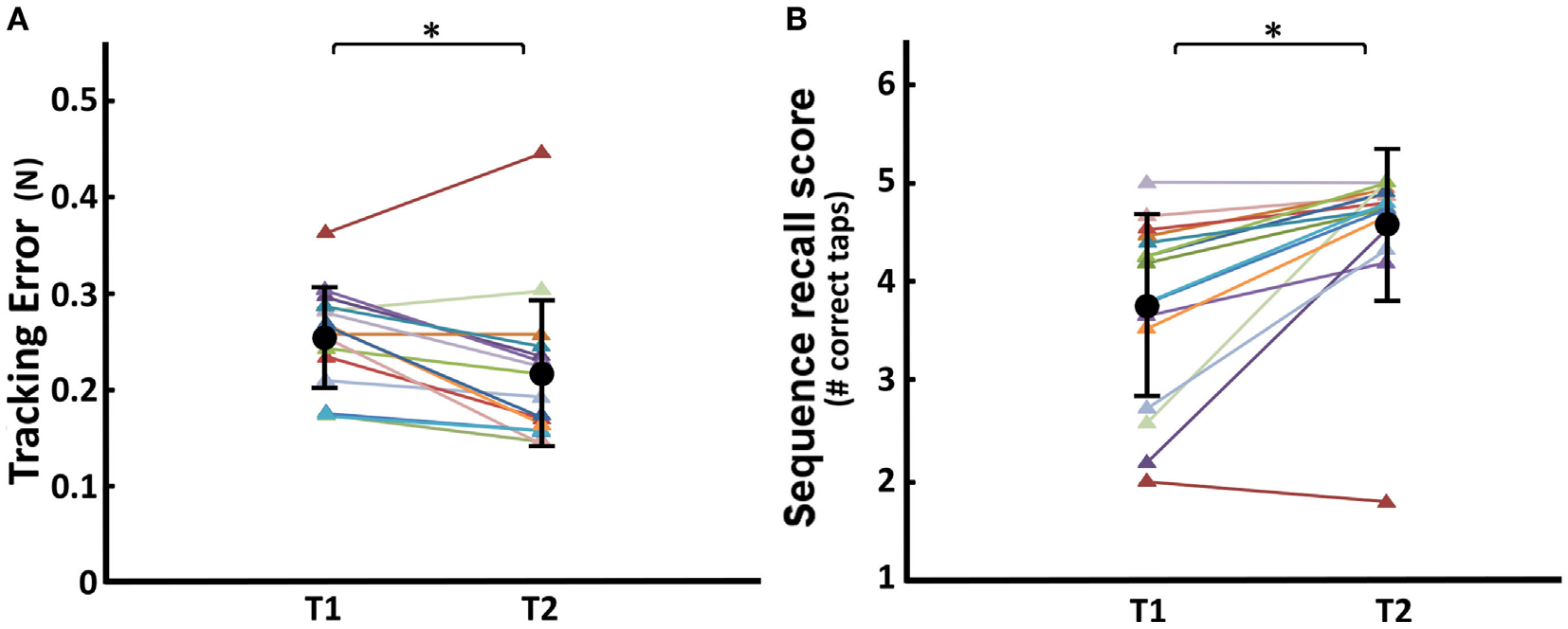
Comparison of pre- and post-remediation performance in dexterous control. **(A)** Finger Force Manipulandum (FFM) tracking error. **(B)** FFM sequence recall score. T1, pre-remediation; T2, post-remediation. Group average ± SD in black circle and bars, respectively. Colored triangles and lines represent individual scores (*N* = 15) across each condition. *Significant difference T1 vs. T2 at *P* < 0.05.

In the post-remediation condition, ANOVA showed a significantly decreased ramp error during force tracking (T1/T2 effect: *F* = 8.86, *P* = 0.009; Figure 7A), and also a significantly increased sequence recall score during sequential finger tapping (T1/T2 effect: *F* = 10.26, *P* = 0.005; Figure 7B). Remediation did not lead to significant changes in tap interval variability or degree of finger individuation.

Changes in clinical and neuropsychological scores following remediation are listed in Table 2. In particular, PANSS total score (Wilcoxon *Z* = 2.95, *P* = 0.003) and sub-scores (Negative: *Z* = 2.73, *P* = 0.006; General: *Z* = 2.73, *P* = 0.006; Disorganization: *Z* = 2.86, *P* = 0.004) decreased significantly after remediation, except for the Positive subscale. The total NSS score also decreased significantly after remediation (*Z* = 2.04, *P* = 0.04) but not the sub-scores. Among the neuropsychological scores, those related to attention (D2, Stroop) improved significantly.

## Discussion

Manual dexterity, quantified by four behavioral components extracted from four visuomotor tasks, was significantly affected in stabilized patients with schizophrenia. In the patient group, each component was significantly impaired compared to control subjects. This concerned (i) lower force-tracking accuracy, (ii) higher variability during repetitive finger tapping, (iii) more errors during memorized finger sequences, and (iv) lower degree of finger individuation. Patients showed individually different profiles of deficient manual dexterity. A composite performance measure discriminated patients with schizophrenia from control subjects with a sensitivity of 79% and a specificity of 90%. Only tracking error and degree of individuation correlated with NSS scores, suggesting that together these four components capture new and complementary information on manual dexterity in schizophrenia.

### Affected Dexterity Components: Correlation to Clinical Status and Medication

In terms of *force tracking*, which requires visuomotor matching and fine on-line adjustments of low finger forces, clear deficits were found in patients with schizophrenia: they produced about 40% more error than controls, similar to deficits found in controlling power grip forces (30) and consistent with less accurate control of steady grip (32) and finger flexion force (28). However, grasp function (evaluated by the ratio of grip/load force) was not or only marginally affected in schizophrenia (37, 63), and when affected, it was considered a side effect of antipsychotic medication (10). There is thus clear evidence for a deficit in voluntary and visually controlled force modulation, but less so for force control during grasp. Visuomotor force tracking requires real-time sensorimotor mapping and integration, and the patients’ precision in this task correlated positively with NSS sub-scores in motor coordination, motor integration and sensory integration. This validates that these NSS sub-scores reflect lack of precision (clumsiness) in tasks requiring sensorimotor integration. Interestingly, visuomotor precision (ramp error) was the only key-FFM score to correlate with antipsychotic medication (CPZe).

The *sequential finger-tapping* task required successive activation of fingers to produce movement sequences in the context of procedural learning: patients with schizophrenia performed significantly less well than controls, in particular during recall. Nonetheless, during learning patients reached a level similar to control subjects, suggesting that altered memory processing and not learning might be the cause of this deficit. These data agree with previous results on motor sequence learning involving finger-thumb opposition in schizophrenia (64). However, motor sequencing is more generally affected, as shown by studies on grip control (38), planned grasping (63), and other tasks (e.g., NSS (65)). Sequence recall correlated with the PANSS (global and disorganization sub-score). This (rather weak) association (in line with (66)) might not be surprising, since cognitive aspects contribute far more to PANSS scores than sensorimotor components or deficient dexterity (67). The lack of correlations between the FFM recall score and working memory scores (WAIS-III) might be related to differences between cognitive and procedural learning (68, 69).

The *finger-tapping* task required primarily temporal motor coordination. Deficits in finger-tapping tasks, such as reduced maximal tapping speed [(18, 68, 70, 71), but not in Ref. (72)], higher than required tapping frequency (73), and higher tapping variability (5, 73) have been previously shown. We found increased tapping variability, but not increased tapping frequency, whether cued or not (most likely due to our more constrained task conditions). Finger-tapping performance has been considered as a potential endophenotype, with a typical performance gradient that increased from patients, to unaffected relatives and finally to control subjects (18, 70, 71). However, timing, as assessed with the FFM task, did not correlate to any of the clinical/neuropsychological tests or to antipsychotic medication.

*The degree of finger individuation* (assessed during multi-finger tapping) was significantly lower in patients with schizophrenia. Although finger individuation is a key feature of manual dexterity (74), to our knowledge, this has not been previously quantified in schizophrenia. However, the Purdue Pegboard test, which requires some (non-quantifiable) degree of finger independence, has repeatedly been used and might be compared to our degree of finger individuation: schizophrenia patients showed lower Pegboard scores than controls (31, 39, 72) and deficient scores were related to social functioning (38). In our case, degree of finger individuation correlated with sensory integration (NSS sub-score), with attention (D2 score), and with working memory (WAIS score). Tentatively, this may be interpreted as common cognitive operations involved in task-related finger individuation, requiring (i) visuomotor mapping (NSS) to determine the digits to be moved (and those not to), (ii) working memory (WAIS-III) to maintain this mapping, and (iii) focused attention (D2) since targets changed unpredictably. The lower scores in these basic cognitive operations may (in part) explain the resulting deficit in finger individuation, and are in line with neurocognitive deficits observed in schizophrenia (1, 75). This does not exclude that the motor command itself or its transmission *via* the corticospinal tract may be perturbed (76, 77).

### Limitations of the Study

Our sample of stabilized patients may have masked even stronger deficits in dexterity, likely to be present in more severely affected (refractory or acute phase) patients with schizophrenia (78). A larger sample may also permit identification of subgroups of dexterity profiles and investigation of their relation to neuropsychological/clinical subtypes [e.g., Ref. (79)]. The absence of drug-naïve patients prevented us from providing direct evidence against an antipsychotic medication effect on the dexterity scores. There is, however, indirect evidence (see below) and evidence from other studies against this assumption (28–31). Similar limitations (plus a significant dropout rate) affected the remediation protocol. Some of these issues and others, such as the specificity of deficient dexterity components, their potential use as trait or state markers, or their occurrence in “high risk” populations, might be addressed in future studies.

### Can Antipsychotic Medication Explain Impaired Dexterity?

A number of findings speak against antipsychotic medication as a main cause of deficient dexterity in schizophrenia. First, ramp error decreased after cognitive remediation, while antipsychotic medication (CPZe) remained constant. Second, CPZe only correlated with ramp error but not with motor sequence recall, timing, or independence of finger movements. Third, the observed correlations between these three dexterity scores and clinical/neuropsychological scores (Table 3) were robust when adding CPZe as a covariate. This is consistent with previous studies on upper limb deficits vs. antipsychotic medication in schizophrenia (28–31).

### Individual Differences in Dexterity Profiles: Toward a Useful Clinical Marker?

Our results provide quantitative evidence at the group level that dexterity is affected in schizophrenia, that four different components of dexterity can be distinguished, that each is significantly affected, and that these components are largely independent of each other. Whether the heterogeneous profiles at the individual level will cluster into subtypes and correlate with clinical phenotypes in schizophrenia [e.g., Ref. (79)] needs further investigation. Discrimination between patients and controls using ROC showed that affected manual dexterity provided a measure with a sensitivity of 79% and a specificity of 90%. Thus dexterity, quantified with FFM, showed a better discrimination than total NSS score and showed similar predictive accuracy compared to gaze deficits (80, 81). Therefore, manual dexterity may well be considered a potential clinical marker in schizophrenia.

### Responsiveness to Cognitive Remediation

After cognitive remediation patients showed an improvement in main clinical and neuropsychological scores. Moreover, patients also improved significantly in two of the four main FFM scores, suggesting that these measures are sensitive enough to detect small variations in manual dexterity, only in part captured by NSS. Crucially, the remediation protocol did not include any FFM training, but used more general aspects of sensorimotor training. This suggests that tracking error and sequence recall, which varied as a function of remediation, may be more state-related [similar to NSS (82)], whereas degree of individuation and tap variability, remediation invariant, may be considered potential trait markers.

In conclusion, our results are consistent with deficits in manual dexterity being a genuine phenotype of the underlying pathophysiology in schizophrenia. Our data suggest that quantitative assessment of these dysfunctions may serve as useful clinical markers in schizophrenia.

## Ethics Statement

The study, approved by the local ethics committee (study no. 2011-A00454-37), complied with the Declaration of Helsinki. Subjects provided written informed consent.

## Author Contributions

MT collected data and performed statistical analysis and drafted first version of manuscript. LC performed some data analysis and contributed to the Section “Material and Methods” part of this manuscript. LB-H and MC collected data on neuropsychological performance of the patients and contributed to the methods and discussion parts about these measures. IA was responsible for study design, recruitment and the clinical assessments, and editing of full manuscript. PL, M-OK, and MM contributed to the conception of this protocol, interpreted results, and edited the manuscript.

## Conflict of Interest Statement

MM and PL have a patent on the method for multidimensional measurement of manual dexterity (EP2659835A1) but do not own the commercialization rights. The other authors report no financial interests or potential conflicts of interest.

## Footnotes

^1^www.sensix.fr.

^2^www.ced.co.uk.

^3^http://www.statsoft.com/.
